# Supplementary material for: Establishing a dementia care competency framework for care partners, health and social care providers: A modified Delphi study protocol
Source: PLoS One. 2025 Nov 10;20(11):e0336566. doi: 10.1371/journal.pone.0336566 (PMC12599962; doi:10.1371/journal.pone.0336566)
Supplement: S1 Table — (DOCX) [file pone.0336566.s001.docx]

**S1 Table. Completed reporting checklist**

| Item No. | Section | Checklist Item | Page No. |
| --- | --- | --- | --- |
| T1 | Title | Identify the article as reporting a consensus exercise and state the consensus methods used in the title. | 1 |
| I1 | Introduction | Explain why a consensus exercise was chosen over other approaches. | 5-6 |
| I2 |  | State the aim of the consensus exercise, including its intended audience and geographical scope (national, regional, global). | 5 |
| I3 |  | If the consensus exercise is an update of an existing document, state why an update is needed, and provide the citation for the original document. | N/A |
| M1 | Methods  Registration | If the study or study protocol was prospectively registered, state the registration platform and provide a link. If the exercise was not registered, this should be stated. | N/A |
| M2 | Selection of SC and/or panellists | Describe the role(s) and areas of expertise or experience of those directing the consensus exercise. | 6-7 |
| M3 |  | Explain the criteria for panellist inclusion and the rationale for panellist numbers. State who was responsible for panellist selection. | 6-7 |
| M4 |  | Describe the recruitment process (how panellists were invited to participate). | 7-8 |
| M5 |  | Describe the role of any members of the public, patients or carers in the different steps of the study. | 6-8, 13 |
| M6 | Preparatory research | Describe how information was obtained prior to generating items or other materials used during the consensus exercise. | 8, 13 |
| M7 |  | Describe any systematic literature search in detail, including the search strategy and dates of search or the citation if published already. | N/A |
| M8 |  | Describe how any existing scientific evidence was summarised and if this evidence was provided to the panellists. | 8, 13 |
| M9 | Assessing consensus | Describe the methods used and steps taken to gather panellist input and reach consensus (for example, Delphi, RAND-UCLA, nominal group technique). | 9-11 |
| M10 |  | Describe how each question or statement was presented and the response options. State whether panellists were able to or required to explain their responses, and whether they could propose new items. | 8-10 |
| M11 |  | State the objective of each consensus step. | 9-10 |
| M12 |  | State the definition of consensus (for example, number, percentage, or categorical rating, such as ‘agree’ or ‘strongly agree’) and explain the rationale for that definition. | 10-12 |
| M13 |  | State whether items that met the prespecified definition of consensus were included in any subsequent voting rounds. | 11-12 |
| M14 |  | For each step, describe how responses were collected, and whether responses were collected in a group setting or individually. | 9-10 |
| M15 |  | Describe how responses were processed and/or synthesised. | 9-10 |
| M16 |  | Describe any piloting of the study materials and/or survey instruments. | 9 |
| M17 |  | If applicable, describe how feedback was provided to panellists at the end of each consensus step or meeting. | 12-13 |
| M18 |  | State whether anonymity was planned in the study design. Explain where and to whom it was applied and what methods were used to guarantee anonymity. | 12 |
| M19 |  | State if the steering committee was involved in the decisions made by the consensus panel. | 7 |
| M20 | Participation | Describe any incentives used to encourage responses or participation in the consensus process. | 7 |
| M21 |  | Describe any adaptations to make the surveys/meetings more accessible. | 7 |
| R1 | Results | State when the consensus exercise was conducted. List the date of initiation and the time taken to complete each consensus step, analysis, and any extensions or delays in the analysis. | 12-13 |
| R2 |  | Explain any deviations from the study protocol, and why these were necessary. | N/A |
| R3 |  | For each step, report quantitative (number of panellists, response rate) and qualitative (relevant socio-demographics) data to describe the participating panellists. | N/A |
| R4 |  | Report the final outcome of the consensus process as qualitative (for example, aggregated themes from comments) and/or quantitative (for example, summary statistics, score means, medians and/or ranges) data. | N/A |
| R5 |  | List any items or topics that were modified or removed during the consensus process. Include why and when in the process they were modified or removed. | N/A |
| D1 | Discussion | Discuss the methodological strengths and limitations of the consensus exercise.  *Include factors that may have impacted the decisions (for example, response rates, representativeness of the panel, potential for feedback during consensus to bias responses, potential impact of any non-anonymised interactions).* | 14 |
| D2 |  | Discuss whether the recommendations are consistent with any pre-existing literature and, if not, propose reasons why this process may have arrived at alternative conclusions. | N/A |
| O1 | Other information | List any endorsing organisations involved and their role. | 7 |
| O2 |  | State any potential conflicts of interests, including among those directing the consensus study and panellists. Describe how conflicts of interest were managed. | 2 |
| O3 |  | State any funding received and the role of the funder. | 2 |
